# Serum Non-high-density lipoprotein cholesterol concentration and risk of death from cardiovascular diseases among U.S. adults with diagnosed diabetes: the Third National Health and Nutrition Examination Survey linked mortality study

**DOI:** 10.1186/1475-2840-10-46

**Published:** 2011-05-23

**Authors:** Chaoyang Li, Earl S Ford, James Tsai, Guixiang Zhao, Lina S Balluz, Samuel S Gidding

**Affiliations:** 1 Division of Behavioral Surveillance, Centers for Disease Control and Prevention, Atlanta, GA, USA; 2 Division of Adult and Community Health, Centers for Disease Control and Prevention, Atlanta, GA, USA; 3 Division of Blood Disorders, Centers for Disease Control and Prevention, Atlanta, GA, USA; 4 Nemours Cardiac Center, A. I. DuPont Hospital for Children, Wilmington, DE, USA

**Keywords:** lipids, lipoproteins, mortality, diabetes mellitus, cardiovascular diseases

## Abstract

**Background:**

Non-high-density lipoprotein cholesterol (non-HDL-C) measures all atherogenic apolipoprotein B-containing lipoproteins and predicts risk of cardiovascular diseases (CVD). The association of non-HDL-C with risk of death from CVD in diabetes is not well understood. This study assessed the hypothesis that, among adults with diabetes, non-HDL-C may be related to the risk of death from CVD.

**Methods:**

We analyzed data from 1,122 adults aged 20 years and older with diagnosed diabetes who participated in the Third National Health and Nutrition Examination Survey linked mortality study (299 deaths from CVD according to underlying cause of death; median follow-up length, 12.4 years).

**Results:**

Compared to participants with serum non-HDL-C concentrations of 35 to 129 mg/dL, those with higher serum levels had a higher risk of death from total CVD: the RRs were 1.34 (95% CI: 0.75-2.39) and 2.25 (95% CI: 1.30-3.91) for non-HDL-C concentrations of 130-189 mg/dL and 190-403 mg/dL, respectively (*P *= 0.003 for linear trend) after adjustment for demographic characteristics and selected risk factors. In subgroup analyses, significant linear trends were identified for the risk of death from ischemic heart disease: the RRs were 1.59 (95% CI: 0.76-3.32) and 2.50 (95% CI: 1.28-4.89) (*P *= 0.006 for linear trend), and stroke: the RRs were 3.37 (95% CI: 0.95-11.90) and 5.81 (95% CI: 1.96-17.25) (*P *= 0.001 for linear trend).

**Conclusions:**

In diabetics, higher serum non-HDL-C concentrations were significantly associated with increased risk of death from CVD. Our prospective data support the notion that reducing serum non-HDL-C concentrations may be beneficial in the prevention of excess death from CVD among affected adults.

## Introduction

Non-high-density lipoprotein cholesterol (non-HDL-C) concentration is a composite marker of several atherogenic lipoproteins, including low-density lipoprotein (LDL), very-low-density lipoprotein (VLDL), intermediate-density lipoprotein (IDL), and lipoprotein (a) [1]. Non-HDL-C concentration can be measured by subtracting high-density lipoprotein cholesterol (HDL-C) concentration from total serum cholesterol concentration and it may be used as a candidate biometrical equivalent to apolipoprotein B100 in diabetes [2]. Serum total cholesterol and LDL cholesterol have been used as major laboratory measures in clinical practice to assess cardiovascular risk in the general population and disease management and prognosis in patients [1]. Recent studies, however, have shown that non-HDL-C concentration is similar to or better than LDL-C alone in the prediction of cardiovascular disease (CVD) incidence and mortality in the general population [3-7] and in the prediction of CVD incidence among people with type 2 diabetes [8-10].

People with diabetes have an excess risk of incidence and mortality from CVD [11,12], however whether non-HDL-C concentration predicts CVD mortality among people with diabetes is not well understood. A few previous studies have assessed the associations of non-HDL-C with risk of death from coronary heart disease (CHD) [13] and CVD [14], but reported conflicting results. Demographic differences in study cohorts, small sample sizes, or short follow-up periods may be attributable to inconsistency in previous study findings. To determine the association between serum non-HDL-C concentration and risk of death from CVD in a larger, more racially diverse cohort of diabetic participants over a longer study period, we analyzed baseline data from the Third National Health and Nutrition Examination Survey (NHANES III) of U.S. adults aged 20 years and older and the linked mortality data on NHANES III participants.

## Methods

### Study population

The NHANES III (1988-1994) survey recruited study participants using a multistage, stratified sampling process designed to produce a survey sample representative of the civilian non-institutionalized U.S. population [15]. The NHANES III survey underwent Institutional Review Board approval, and written informed consent was received from all participants. After being interviewed at their homes, participants were invited to a mobile examination center where they had a clinical examination and provided a blood sample. Response rates were 86% for the household interviews and 78% for the medical examinations [16]. Of 1,262 survey participants who had diagnosed diabetes and attended a mobile examination center, 1,179 (93.4%) provided serum samples for non-HDL-C measurements. After exclusion of participants who had missing data on covariates (n = 57), our study cohort consisted of 1,122 NHANES III participants (88.9% of all participants who attended a mobile examination center).

### Diabetes status and duration

Diagnosed diabetes was ascertained by asking participants, "Have you ever been told by a doctor that you have diabetes?" Responses were coded as "yes," "yes, but female told only during pregnancy," or "no." A diabetes history restricted to pregnancy was coded as "no" diabetes. Diabetes duration in years was determined by subtracting age at diagnosis of diabetes from age at baseline examination.

### Laboratory procedures

A detailed description of laboratory procedures has been published elsewhere [17]. In brief, serum samples were aliquoted, stored at -70°C, and shipped on dry ice to the NHANES laboratory at the Centers for Disease Control and Prevention. Total serum cholesterol was measured enzymatically in a series of coupled reactions that hydrolyze cholesterol esters and oxidize the 3-OH cholesterol group. HDL-C concentrations were measured after the precipitation of the other lipoproteins with a polyanion/divalent cation mixture. Both total cholesterol and HDL-C analyses were performed with the Hitachi 704 Analyzer (Boehringer Mannheim Diagnostics, Indianapolis, Indiana). Triglyceride concentrations were measured enzymatically in serum after specimens were hydrolyzed to glycerol through a series of coupled reactions. Serum non-HDL-C concentrations were calculated by subtracting HDL-C concentrations from total cholesterol concentrations. Serum LDL-C concentration were estimated from measured values of total cholesterol, triglycerides, and HDL-C according to the Friedewald formula (i.e., *C*_LDL-C _= *C*_Total cholesterol _- *C*_HDL-C _- Triglycerides/5) among participants with triglycerides ≤ 400 mg/dL [17,18].

C-reactive protein (CRP) was measured at the Department of Laboratory Medicine, University of Washington, by using latex-enhanced nephelometry (Behring Diagnostics, Inc., Somerville, New Jersey). The continuum of CRP concentrations was categorized to two groups: < 3.0 mg/L and ≥ 3.0 mg/L. The concentrations of glycated hemoglobin A1c (% ) were measured at the Diabetes Diagnostic Laboratory at the University of Missouri, Columbia, Missouri by using the ion-exchange, high-performance liquid chromatography on the Diamat Analyzer System (Bio-Rad Laboratories, Hercules, California). Serum creatinine concentrations were measured at the White Sands Research Center (Coulston Foundation) laboratory (Alamogordo, New Mexico) by using the kinetic alkaline picrate reaction method on a Roche/Hitachi 737 analyzer (Roche Diagnostics, Indianapolis, Indiana). Glomerular filtration rate (GFR) was estimated with the new equation developed by the Chronic Kidney Disease Epidemiology Collaboration (CKD-EPI) on the basis of standardized serum creatinine level (standard creatinine = - 0.184 + 0.960 × NHANES III uncalibrated serum creatinine) [19,20]. Estimated GFR is reported in ml/minute per 1.73 m^2 ^of body surface area.

### Demographic characteristics and physical examination measures at baseline

Participants' age (years), race/ethnicity (non-Hispanic white, non-Hispanic black, Mexican American, and other race/ethnicity), education level (less than high school, high school, and beyond high school), smoking status (current smoker, former smoker, and never smoked), alcohol consumption (times in the previous month), and use of prescribed medicine to lower blood cholesterol (yes, no) were determined by self reports of participants. Physical activity level was determined by participants' self-reported frequency of engaging in specific types of leisure-time exercise or activities during the previous month multiplied by the rate of energy expenditure (intensity rating) for those activities according to a standardized coding method [21]. Participants' body mass index (BMI; weight [kg]/height^2 ^[m]) was calculated from their measured weight and height. The mean blood pressure was calculated as the average of the second and third readings for those who had three measurements, the second reading for those who had two measurements, and the only reading for those who had one measurement [22].

### Linked mortality data through 2006

The NHANES III participants with sufficient identifying data were matched to the National Death Index (NDI) to determine their survival status through 2006 [23]. The National Center for Health Statistics (NCHS) performed linkage with probabilistic matching between NHANES III and NDI data and reviewed death certificates to ensure that deaths reported in the NDI were matched to the correct NHANES III participants. We used a standardized list of 113 causes based on International Classification of Diseases, Tenth Revision (ICD-10) codes to determine decedents' underlying cause of death [24]. We divided CVD into the subcategories of ischemic heart disease (IHD), stroke, and other CVD.

### Statistical analysis

We first examined the population distribution of serum non-HDL-C concentrations by sex and age. We divided the continuum of serum non-HDL-C concentrations into three categories (<130, 130-189, and ≥ 190 mg/dL) according to the Third Report of the National Cholesterol Education Program Expert Panel on Detection, Evaluation, and Treatment of High Blood Cholesterol in Adults (NCEP ATP III) [25]. We compared baseline demographic characteristics and cardiovascular risk factors of participants according to these three levels using 2-sample *t *tests.

We conducted Cox proportional hazard regression analysis to estimate relative risks (hazard ratios) and 95% confidence interval (CI) for death from total CVD, IHD, stroke, and other CVD in which we treated serum non-HDL-C as a categorical variable with 3 levels. We used age in months as the time scale in survival analyses with left truncation (i.e., participants entered into the cohort at different ages). Participants' age in months at the end of follow-up was determined by adding their age in months at baseline examination to the number of months they were followed. We provided unadjusted risk estimates and adjusted risk estimates by controlling for the selected potential confounders. We used *t *tests with orthogonal polynomial coefficients to test for linear trends in relative risks in the Cox regression models. To account for the possible nonlinear association between serum non-HDL-C concentrations and risk of death from CVD, we also estimated relative risks by performing Cox proportional hazard regression analyses using serum non-HDL-C concentrations as a continuous variable and adding its cubic regression spline term with three knots [26].

To further examine whether the association between serum non-HDL-C concentration and risk of death from CVD may be consistent in sub-populations, we conducted subgroup analyses using a 10 mg/dL increase in non-HDL-C as the predictor variable, stratified by demographic characteristics and selected CVD risk factors. To ensure adequate sample size in each level of stratification variables, we dichotomized the following stratification variables: age (20-64 y versus 65 y), race (non-Hispanic white versus non-white), educational attainment (<high school versus high school or beyond), diabetes duration (<10 y versus ≥ 10 y), follow-up length (<10 y versus ≥ 10 y), smoking status (current or former smoker versus never smoked), We used Wald χ^2 ^tests to assess the interactions between serum non-HDL-C concentrations and all stratification variables on the risk of death from IHD.

We conducted sensitivity analyses by replicating the main analyses using an alternative definition of diabetes based on both self-reported diabetes diagnosis and glycated hemoglobin A1C level ≥ 6.5% among adults without a diagnosis of diabetes. We also compared the relative risk of death from CVD between non-HDL-C and LDL-C, using a restricted sample of adults who attended the morning session of a mobile examination center, had fasted at least 8 hours, and had a valid LDL-C value (n = 366).

Results of 2-tailed *t *tests used in two groups or χ^2 ^tests used in 2-way comparisons were considered to be statistically significant if *P *values were less than 0.05 and estimates of relative risks were significantly different from 1 if the 95% CI did not include 1. Data were analyzed using the SAS System for Windows (Release 9.2) (SAS Institute Inc., Cary, North Carolina) and SUDAAN software (Release 10, Research Triangle Institute, Research Triangle Park, NC). In all analyses, sample weights were used to account for the varying probabilities of complex sampling design and nonresponse and produce nationally representative estimates.

## Results

Of the 1,122 eligible NHANES III adult participants with diagnosed diabetes and with no missing data on all selected variables in our study, 655 were found to have died over a median follow-up period of 12.4 years (Table 1). Of those who died from all causes, 299 died from CVD (46.4%; including 182 deaths from IHD, 50 deaths from stroke, and 67 deaths from other CVD).

**Table 1:** Number of Deaths from CVD among Adults Aged ≥ 20 Years with Diagnosed Diabetes in the United States, the Third National Health and Nutrition Examination Survey Linked Mortality Study, 1988-2006

Cause of Death	**List of 113 Causes of Death**^**a**^	No. and Rate of Death (1/1,000 person-year)	Total Cohort (N = 1,122)	**Serum non-HDL-C Concentration (mg/dL)**^**b**^		
				
				35-129	130-189	190-403
Person-years (sample)			11,807	1,915	6,222	3,670
Person-years (weighted)			89,423,467	14,290,472	45,912,850	29,220,145
All causes	1-135	No. (sample)	655	125	303	227
		No. (weighted)	4,197,413	601,058	1,906,185	1,690,170
		Rate (weighted)	46.9	42.1	41.5	57.8
Total CVD	53-74	No. (sample)	299	50	137	112
		No. (weighted)	1,946,876	217,843	819,108	909,926
		Rate (weighted)	21.8	15.2	17.8	31.1
IHD	58-63	No. (sample)	182	26	90	66
		No. (weighted)	1,225,668	124,344	531,179	570,144
		Rate (weighted)	13.7	8.7	11.6	19.5
Stroke	70	No. (sample)	50	9	20	21
		No. (weighted)	262,765	11,748	121,332	129,685
		Rate (weighted)	2.9	0.8	2.6	4.4
Other CVD	53-57, 64-69, 71-74	No. (sample)	67^c^	15	27	25
		No. (weighted)	458,443	81,750	166,597	210,096
		Rate (weighted)	5.1	5.7	3.6	7.2

Abbreviations: CVD, cardiovascular disease; IHD, ischemic heart disease; non-HDL-C, non-high-density lipoprotein cholesterol.

^a ^The list of 113 causes of death was developed based on ICD-10 codes [24].

^b ^To convert HDL-C or non-HDL-C concentrations in mg/dL to concentrations in mmol/L, multiply by 0.02586.

^c ^Include 3 cases of hypertensive heart disease, 1 hypertensive heart and renal disease, 18 cases of heart failure, 30 cases of all other forms of heart disease, 5 cases of essential (primary) hypertension and hypertensive renal disease, 5 cases of atherosclerosis, 1 case of aortic aneurysm and dissection, and 4 cases of other diseases of arteries, arterioles and capillaries.

The mean concentration of serum non-HDL-C was 174.25 mg/dL (±2.89 mg/dL) for the entire cohort, including 165.45 mg/dL (±0.4.40 mg/dL) among men, and 182.02 mg/dL (±2.69 mg/dL) among women (*P *< 0.001 for difference by sex). We found significant differences by serum non-HDL-C concentration levels in the following characteristics: mean body mass index, systolic blood pressure, serum HDL-C concentration, distribution of study participants by sex, CRP concentration ≥ 3.0 mg/L, and use of prescribed medicine to lower blood cholesterol (all *P *< 0.05) (Table 2). Men had a higher rate of using prescribed medicine to lower blood cholesterol than women (10.4% vs. 7.7%, *P *= 0.38), albeit the difference in the rates did not reach statistical significance.

**Table 2:** Baseline Characteristics of Adults Aged ≥ 20 Years with Diabetes in the Total Cohort and According to Serum Non-HDL-C Concentrations, NHANES III (1988-1994)

Variable	Total cohort	**Serum non-HDL-C concentration (mg/dL)**^**a**^			
			
		35-129	130-189	190-403	***P*-value**^**b**^
Unweighted sample size	1,122	200	563	359	
Weighted %	100.0 ± 0.0^c^	16.0 ± 2.1	49.2 ± 2.6	34.8 ± 2.7	
Follow-up length (yr)	12.4 ± 0.3	12.9 ± 1.2	12.5 ± 0.3	11.9 ± 0.8	
Serum non-HDL-C concentration (mg/dL)	174.2 ± 2.9	106.7 ± 3.5	160.2 ± 1.0	225.1 ± 2.6	<0.001
**Demographic characteristics**					
Age (yr)	60.3 ± 0.7	58.4 ± 3.1	60.2 ± 1.0	61.2 ± 0.9	0.39
Male (%)	46.9 ± 2.5	60.9 ± 7.1	49.8 ± 3.7	36.4 ± 3.7	0.006
Non-Hispanic white (%)	74.9 ± 2.0	68.9 ± 5.5	74.4 ± 2.5	78.3 ± 2.9	0.09
Less than high school (%)	42.6 ± 3.0	38.5 ± 6.6	39.8 ± 4.2	48.5 ± 4.0	0.21
Diabetes duration (yr)	9.4 ± 0.4	10.5 ± 1.0	9.0 ± 0.5	9.3 ± 0.6	0.30
**CVD risk factors**					
Current or former smokers (%)	58.9 ± 2.4	58.1 ± 8.0	60.2 ± 3.2	57.5 ± 3.9	0.94
Alcohol consumption (times in previous month)	4.5 ± 0.7	5.9 ± 1.9	4.7 ± 1.2	3.5 ± 1.0	0.26
Body mass index (kg/m^2^)	30.1 ± 0.3	27.6 ± 0.6	30.3 ± 0.4	30.9 ± 0.5	<0.001
Leisure-time physical activity (METS)	80.6 ± 6.5	105.4 ± 19.5	81.0 ± 8.3	68.7 ± 8.8	0.10
Systolic blood pressure (mm Hg)	134.0 ± 1.1	129.0 ± 2.4	132.6 ± 1.6	138.3 ± 2.0	0.007
Serum HDL-C (mg/dL)	45.7 ± 0.8	53.1 ± 2.4	45.6 ± 1.0	42.4 ± 1.0	<0.001
Glomerular filtration rate (mL/min per 1.73 m^2^)	83.5 ± 1.2	85.9 ± 5.7	86.4 ± 1.3	78.3 ± 1.7	0.20
Serum glycated hemoglobin A1C (%)	7.7 ± 0.1	7.4 ± 0.2	7.6 ± 0.1	7.9 ± 0.2	0.06
C-reactive protein ≥ 3.0 mg/L (%)	50.4 ± 3.4	35.6 ± 6.5	50.0 ± 4.1	57.8 ± 4.4	0.003
**Use of prescribed medicine to lower blood cholesterol (%)**	9.0 ± 1.3	4.2 ± 1.9	9.1 ± 2.3	11.1 ± 2.7	0.036

Abbreviations: non-HDL-C, non-high-density lipoprotein cholesterol; HDL-C, high-density lipoprotein; METS, metabolic equivalents.

^a ^To convert HDL-C or non-HDL-C concentrations in mg/dL to concentrations in mmol/L, multiply by 0.02586.

^b ^P-values were obtained from *t *tests for linear trends in the proportions or means across the three non-HDL-C levels.

^c ^All data are weighted percentage ± SE for categorical variables and weighted mean ± SE for continuous variables except for follow-up length (weighted median ± SE).

We found a significant trend in the unadjusted relative risk of death from total CVD, IHD, and stroke (all *P *<0.01 for linear trend) (Table 3). After adjusting for the demographic characteristics and CVD risk factors presented in Table 2, the significant association persisted (all *P *<0.01 for linear trend). No significant association was found between serum non-HDL-C concentration and risk of death from other CVD.

**Table 3:** Estimated Relative Risk (Hazard Ratio) of Death from CVD Among U.S. Adults Aged ≥ 20 Years with Diabetes According to Serum Non-HDL-C Concentrations, the Third National Health and Nutrition Examination Survey Linked Mortality Study, 1988-2006

Cause of death	**Serum non-HDL-C concentration (mg/dL)**^**a**^			
		
	35-129	130-189	190-403	**P-value**^**b**^
Unadjusted risk estimates				
Total CVD	1 [Ref]	1.19 (0.71-1.98)	2.19 (1.38- 3.47)	<0.001
IHD	1 [Ref]	1.36 (0.71-2.58)	2.32 (1.30- 4.15)	0.004
Stroke	1 [Ref]	2.91 (0.89-9.57)	5.34 (1.88-15.16)	0.001
Other CVD	1 [Ref]	0.67 (0.31-1.44)	1.53 (0.78- 3.03)	0.21
Adjusted risk estimates				
Total CVD	1 [Ref]	1.34 (0.75- 2.39)	2.25 (1.30- 3.91)	0.003
IHD	1 [Ref	1.59 (0.76- 3.32)	2.50 (1.28- 4.89)	0.006
Stroke	1 [Ref]	3.37 (0.95-11.90)	5.81 (1.96-17.25)	0.001
Other CVD	1 [Ref]	0.69 (0.25- 1.88)	1.40 (0.55- 3.55)	0.46

Abbreviations: CVD, cardiovascular disease; CI, confidence interval; IHD, ischemic heart disease; non-HDL-C, non-high-density lipoprotein cholesterol; Ref, reference value.

^a ^To convert HDL-C or non-HDL-C concentrations in mg/dL to concentrations in mmol/L, multiply by 0.02586.

^b ^T-tests were used for testing linear trends. Age was the time scale in the Cox proportional hazard regression analyses. Relative risks (hazard ratios) and their 95% CIs were adjusted for sex, race/ethnicity, education attainment, diabetes duration, body mass index, leisure-time physical activity, smoking status, alcohol consumption, systolic blood pressure, serum HDL-C concentration, glomerular filtration rate, C-reactive protein, glycated hemoglobin A1C, and self-reported use of prescribed medicine to lower blood cholesterol.

N = 1,122.

We found an elevated relative risk of death from CVD for each 10 mg/dL increase in serum non-HDL-C concentration in most subgroups stratified by demographic characteristics and CVD risk factors. However, the RR did not reach statistical significance at α = 0.05 level in the following subgroups: women, non-whites, participants with educational attainment of high school or above, adults with a diagnosis of diabetes ≥ 10 years, adults with no leisure-time physical activity, adults with a GFR < 60 mL/min per 1.73 m^2^, adults with a CRP level ≥ 3.0 mg/L, and adults with a serum glycated hemoglobin A1C ≥ 7.0% (*P *values ranging from 0.062 to 0.68) (Table 4). We detected a significant interaction between serum non-HDL-C concentrations and the following variables: sex, age, diabetes duration, follow-up length, alcohol consumption, and systolic blood pressure (all *P *values <0.05).

**Table 4:** Relative Risk (Hazard Ratio) of Deaths from Total CVD Attributable to a 10 mg/dL increase in Serum Non-HDL-C Concentration, Stratified According to Selected Risk Factors, the Third National Health and Nutrition Examination Survey Linked Mortality Study, 1988-2006

Stratification Variable	N (Weighted %)	Non-HDL-C Concentration (Mean ± SE, mg/dL)	**Relative Risk**^**a **^**(95% CI)**	**P Value for Relative Risk Estimate (Interaction)**^**b**^
Total	1,122 (100.0)	174.25 ± 2.89	1.07 (1.04-1.11)	0.0001
Sex				**(0.012)**
Men	501 (46.9)	165.45 ± 4.40*	1.11 (1.05-1.17)	0.0003
Women	621 (53.1)	182.02 ± 2.69	1.04 (0.99-1.09)	0.098
Age (yr)				**(0.032)**
20-64	560 (58.5)	174.03 ± 4.06	1.12 (1.05-1.19)	0.0005
≥ 65	562 (41.5)	174.55 ± 2.79	1.04 (1.01-1.07)	0.019
Race				**(0.091)**
Non-Hispanic white	424 (74.9)	176.01 ± 3.62	1.09 (1.05-1.14)	0.0001
Non-white	698 (25.1)	169.01 ± 3.04	1.01 (0.97-1.05)	0.68
Education				**(0.60)**
≥ High school	429 (57.4)	170.78 ± 4.67	1.06 (0.99-1.13)	0.098
< High school	693 (42.6)	178.91 ± 2.65	1.09 (1.03-1.14)	0.0022
Diabetes duration (yr)				**(0.017)**
< 10	619 (60.7)	172.86 ± 3.86	1.11 (1.06-1.16)	0.0001
≥ 10	503 (39.3)	176.39 ± 3.21	1.02 (0.97-1.07)	0.43
Follow-up length (yr)				**(0.0036)**
< 10	446 (33.4)	177.81 ± 4.81	1.04 (1.01-1.08)	0.014
≥ 10	676 (66.6)	172.46 ± 3.91	1.17 (1.11-1.23)	<0.0001
Smoking status				**(0.67)**
Never smoked	526 (41.1)	174.12 ± 5.46	1.05 (1.00-1.11)	0.045
Current/former smokers	596 (58.9)	174.34 ± 2.26	1.08 (1.02-1.15)	0.015
				
Alcohol consumption				**(0.0077)**
Consumed alcohol	253 (29.1)	164.10 ± 6.33*	1.20 (1.08-1.33)	0.0009
Did not consume alcohol	869 (70.9)	178.42 ± 2.54	1.06 (1.02-1.09)	0.0011
Body mass index (kg/m^2^)				**(0.52)**
< 30	673 (56.7)	166.86 ± 3.96*	1.05 (1.01-1.10)	0.028
≥ 30	449 (43.3)	183.91 ± 2.78	1.10 (1.03-1.17)	0.0038
Leisure-time physical activity				**(0.52)**
Any	745 (73.1)	170.71 ± 3.47*	1.09 (1.04-1.14)	0.0002
None	377 (26.9)	183.87 ± 4.39	1.05 (1.00-1.11)	0.062
Systolic blood pressure (mm Hg)				**(0.030)**
<130	472 (46.2)	167.28 ± 4.97*	1.13 (1.06-1.21)	0.0004
≥130	650 (53.8)	180.24 ± 2.92	1.05 (1.01-1.09)	0.014
Serum HDL-C (mg/dL)				**(0.75)**
Normal	552 (45.6)	162.48 ± 5.09*	1.07 (1.02-1.12)	0.010
Low^c^	570 (54.4)	184.11 ± 3.35	1.07 (1.01-1.14)	0.029
Glomerular filtration rate (mL/min per 1.73 m^2^)				**(0.21)**
≥ 60.0	904 (83.3)	170.89 ± 3.10*	1.10 (1.05-1.16)	0.0002
< 60.0	218 (16.7)	191.03 ± 6.25	1.02 (0.98-1.06)	0.28
C-reactive protein (mg/L)				**(0.13)**
< 3.0	531 (49.6)	167.90 ± 4.89*	1.11 (1.06-1.16)	0.0001
≥ 3.0	591 (50.4)	180.49 ± 3.13	1.03 (0.98-1.09)	0.19
Serum glycated hemoglobin A1C (%)				**(0.44)**
< 7.0	465 (43.1)	167.66 ± 2.78*	1.08 (1.03-1.13)	0.0039
≥ 7.0	657 (56.9)	179.23 ± 4.06	1.06 (0.99-1.12)	0.076

Abbreviations: CVD, cardiovascular disease; CI, confidence interval; IHD, ischemic heart disease; non-HDL-C, non-high-density lipoprotein cholesterol.

^a ^Relative risks (hazard ratios) and their 95% CIs were adjusted for sex, race/ethnicity, education attainment, diabetes duration, body mass index, leisure-time physical activity, smoking status, alcohol consumption, systolic blood pressure, serum HDL-C concentration, glomerular filtration rate, C-reactive protein, glycated hemoglobin A1C, and self-reported use of prescribed medicine to lower blood cholesterol. Age was the time scale in the Cox proportional hazard regression analyses.

^b ^*P *values were based on results of *t *tests for the estimates of hazard ratios within each subgroup and Wald χ^2^-tests for the interaction between serum non-HDL-C concentration and each stratification variable (shown in bold fonts and in parentheses).

^c ^Defined as <40 mg/dL among men and <50 mg/dL among women. To convert HDL-C or non-HDL-C concentrations in mg/dL to concentrations in mmol/L, multiply by 0.02586.

* Within each category of stratification variable, mean value with an asterisk is significantly different from the mean value of another category, *P *= 0.05; *t *tests were used to test for equality.

**Figure 1:**
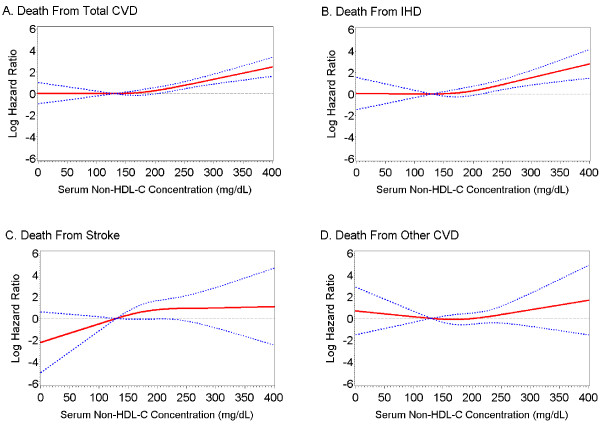
**Estimated relative risk of death among U.S. adults aged 20 years and older with diagnosed diabetes, the NHANES III Linked Mortality Study, 1988-2006**. The red solid line indicates the fitted relationship between the relative risk of death from total CVD (A), IHD (B), stroke (C), and other CVD (D) in relation to the continuum of serum non-HDL-C concentrations with a 3-knot cubic regression spline. The blue dashed lines indicate the 95% confidence intervals surrounding the estimates. Adults with a serum non-HDL-C concentration of 130 mg/dL were set as the reference group. Relative risks (hazard ratios) and their 95% CIs were adjusted for sex, race/ethnicity, education attainment, diabetes duration, body mass index, leisure-time physical activity, smoking status, alcohol consumption, systolic blood pressure, serum HDL-C concentration, glomerular filtration rate, C-reactive protein, glycated hemoglobin A1C, and self-reported use of prescribed medicine to lower blood cholesterol. N = 1,122. To convert non-HDL-C concentration in mg/dL to mmol/L, multiply by 0.02586.N Abbreviations: CVD, cardiovascular disease; IHD, ischemic heart disease; NHANES III, Third National Health and Nutrition Examination Survey; Non-HDL-C, non-high-density lipoprotein cholesterol.

**Figure 2:**
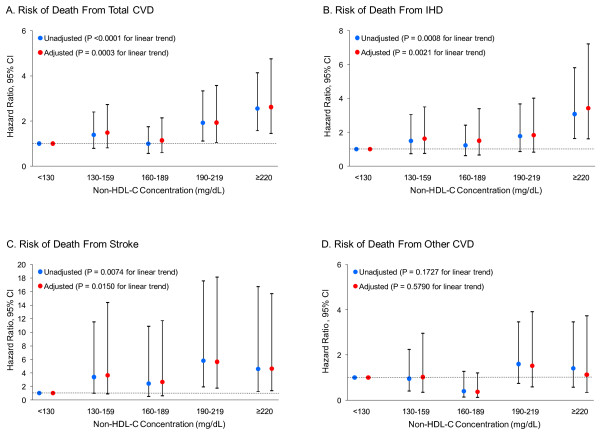
**Hazard ratios of death among U.S. adults aged 20 years and older with diagnosed diabetes, the NHANES III Linked Mortality Study, 1988-2006**. Unadjusted (blue dots) and adjusted (red dots) hazard ratios of death from total CVD (A), IHD (B), stroke (C), and other CVD (D) and their 95% CIs (high-low lines) were adjusted for sex, race/ethnicity, education attainment, diabetes duration, body mass index, leisure-time physical activity, smoking status, alcohol consumption, systolic blood pressure, serum HDL-C concentration, glomerular filtration rate, C-reactive protein, glycated hemoglobin A1C, and self-reported use of prescribed medicine to lower blood cholesterol. Adults with a serum non-HDL-C concentration below 130 mg/dL were set as the reference group. N = 1,122. To convert non-HDL-C concentration in mg/dL to mmol/L, multiply by 0.02586. Abbreviations: CVD, cardiovascular disease; IHD, ischemic heart disease; NHANES III, Third National Health and Nutrition Examination Survey; Non-HDL-C, non-high-density lipoprotein cholesterol.

By modeling non-HDL-C as a continuous variable and adding its cubic regression spline term with 3 knots, we observed a positive and strong dose-response relationship of serum non-HDL-C concentrations to risk of death from total CVD (Figure 1, A), IHD (Figure 1, B), and stroke (Figure 1, C). By modeling non-HDL-C as a categorical variable with five levels, we found that people with a serum non-HDL-C concentration of 190-219 mg/dL or ≥ 220 mg/dL had a significantly elevated risk of death from total CVD (Figure 2, A) and stroke (Figure 2, C). People with a serum non-HDL-C concentration of ≥ 220 mg/dL had a significantly elevated risk of death from IHD (Figure 2, B). We did not detect a significant association between the non-HDL-C concentration and risk of death from other CVD (Figure 1, D and Figure 2, D).

The results of the sensitivity analyses by including adults with undiagnosed diabetes showed that the strengths of the associations between non-HDL-C and risk of death from total CVD, IHD, stroke, and other CVD attenuated slightly, but the patterns and linear trends persisted (Table 5). By using available data of LDL-C among adults who attended the morning session of a mobile examination center, we found that the relative risk of death from total CVD, IHD, stroke, and other CVD for a 10 mg/dl change was similar between non-HDL-C and LDL-C in both unadjusted and adjusted estimates (Table 6).

**Table 5:** Estimated Relative Risk (Hazard Ratio) of Death from CVD Among U.S. Adults Aged ≥20 Years with Both Diagnosed Diabetes and Undiagnosed Diabetes (A1C≥6.5%) According to Serum Non-HDL-C Concentrations, the Third National Health and Nutrition Examination Survey Linked Mortality Study, 1988-2006

Cause of death	Serum non-HDL-C concentration (mg/dL)			
	35-129	130-189	190-403	**P-value**^**a**^
Unadjusted risk estimates				
Total CVD	1 [Ref]	0.93 (0.63-1.38)	1.55 (1.03-2.34)	0.033
Ischemic heart disease	1 [Ref]	0.87 (0.5-1.51)	1.44 (0.87-2.38)	0.14
Stroke	1 [Ref]	3.34 (1.17-9.52)	3.87 (1.54-9.76)	0.0033
Other CVD	1 [Ref]	0.66 (0.32-1.36)	1.42 (0.74-2.71)	0.28
Adjusted risk estimates				
Total CVD	1 [Ref]	0.95 (0.63-1.45)	1.53 (0.95-2.45)	0.072
Ischemic heart disease	1 [Ref]	0.91 (0.50-1.65)	1.51 (0.88-2.59)	0.13
Stroke	1 [Ref]	3.44 (1.23-9.63)	4.33 (1.63-11.49)	0.0026
Other CVD	1 [Ref]	0.58 (0.26-1.31)	1.01 (0.45-2.24)	0.99

Abbreviations: CVD, cardiovascular disease; non-HDL-C, non-high-density lipoprotein cholesterol; Ref, reference value.

^a ^T-tests were used for testing linear trends. Age was the time scale in the Cox proportional hazard regression analyses. Relative risks (hazard ratios) and their 95% CIs were adjusted for sex, race/ethnicity, education attainment, diabetes duration, body mass index, leisure-time physical activity, smoking status, alcohol consumption, systolic blood pressure, serum HDL-C concentration, glomerular filtration rate, C-reactive protein, and self-reported use of prescribed medicine to lower blood cholesterol.

N = 1,647 participants with diagnosed diabetes and undiagnosed diabetes (i.e., glycated hemoglobin A1C ≥ 6.5%).

**Table 6:** Estimated Relative Risk (Hazard Ratio) of Death from CVD Attributable to a 10 mg/dL Increase in Serum Non-HDL-C Concentration and a 10 mg/dL Increase in Serum LDL-C Concentration Among U.S. Adults Aged ≥ 20 Years with Diabetes, the Third National Health and Nutrition Examination Survey Linked Mortality Study, 1988-2006

Cause of death	Non-HDL-C	LDL-C
Unadjusted risk estimates		
Total CVD	1.07 (0.97-1.18)	1.06 (0.95-1.19)
Ischemic heart disease	1.14 (1.01-1.29)	1.14 (0.99-1.32)
Stroke	1.09 (0.93-1.29)	1.08 (0.92-1.27)
Other CVD	0.93 (0.82-1.04)	0.89 (0.78-1.01)
Adjusted risk estimates^a^		
Total CVD	1.07 (0.98-1.16)	1.09 (0.99-1.19)
Ischemic heart disease	1.16 (1.03-1.29)	1.21 (1.05-1.38)
Stroke	1.05 (0.93-1.18)	1.11 (0.94-1.30)
Other CVD	0.89 (0.78-1.02)	0.87 (0.76-1.01)

Abbreviations: CVD, cardiovascular disease; non-HDL-C, non-high-density lipoprotein cholesterol; LDL-C, low-density lipoprotein cholesterol.

^a ^Age was the time scale in the Cox proportional hazard regression analyses. Relative risks (hazard ratios) and their 95% CIs were adjusted for sex, race/ethnicity, education attainment, diabetes duration, body mass index, leisure-time physical activity, smoking status, alcohol consumption, systolic blood pressure, serum HDL-C concentration, glomerular filtration rate, C-reactive protein, glycated hemoglobin A1C, and self-reported use of prescribed medicine to lower blood cholesterol.

N = 366 adults who attended the morning session of a Mobile Examination Center, had fasted at least 8 hours, and had a valid LDL-C value.

## Discussion

In this prospective study of a nationally representative sample of U.S. adults with diagnosed diabetes over a median follow-up length of 12.4 years, we found that higher serum non-HDL-C concentrations were strongly associated with increased risk of death from total CVD, IHD, and stroke. The significant association between a 10 mg/dL increase in serum non-HDL-C concentration and increased risk of death from total CVD was also significant in most subgroups stratified by demographic characteristics and selected CVD risk factors and was similar to that seen for LDL-C.

The strengths of our study include the large sample size and a long follow-up length in a cohort of adult participants with diagnosed diabetes. Studies on the association between non-HDL-C and risk of death from CVD have been scarce and the findings of limited studies are inconsistent. As shown in the study conducted among 313 diabetic patients aged 45 to 64 years in Finland [13], people with a non-HDL-C greater than 5.3 mmol/L (i.e., 204.9 mg/dL) had about 40% increased risk of death from CHD during a 7-year follow-up [13], however the association between non-HDL-C and risk of death from CHD was not statistically significant perhaps due to a low statistical power. Similarly, a recent study failed to identify a significant association between non-HDL-C concentration and risk of mortality from CVD among 1,565 patients with type 2 diabetes during an 11-year follow-up [14]. In contrast to the findings of the previous studies [13,14], the significant and positive associations between serum non-HDL-C concentration and risk of death from CVD as shown in our study suggest that serum non-HDL-C concentration could be an important target in the management of dyslipidemia to reduce CVD mortality among adults with diabetes.

One of the unique results of our study was the strong association between non-HDL-C and risk of death from stroke among adults with diagnosed diabetes. Findings of previous studies with regard to the association between non-HDL-C and risk of incident stroke are mixed among non-diabetic populations. Several studies showed that non-HDL-C was strongly associated with risk of fatal and nonfatal ischemic stroke [27-29], while some other studies showed that non-HDL-C was not related or weakly related to the risk of incident stroke [30-32]. To the best of our knowledge, our study was the first to show the association between non-HDL-C and risk of death from stroke among people with diabetes. These findings have significant clinical implications because people with diabetes have double the risk of stroke incidence and risk of mortality after stroke as seen among people without diabetes [33,34].

While the underlying mechanisms linking non-HDL-C and risk of mortality from IHD and stroke need to elucidated, non-HDL-C concentration could be a marker of atherogenic and pathophysiologic effects of multiple apolipoprotein B-containing lipoproteins and triglyceride-rich lipoproteins including LDL, VLDL, IDL, and lipoprotein (a). Atherosclerosis begins in childhood and its development has been correlated with lipoprotein disorders [35]. Previous studies have shown that non-HDL-C concentration was highly correlated with coronary atherosclerosis measured at autopsy [36]. Our results that non-HDL-C was more strongly associated with risk of death from IHD and stroke than from other CVD suggest that non-HDL-C may play an important role in atherosclerosis and cardioembolism because atherosclerosis is the established underlying pathologic cause of IHD and stroke morbidity and mortality [37,38].

Atherogenic dyslipidemia (i.e., hypertriglyceridemia, low HDL-C concentrations, and elevated small dense LDL) is frequently present among people with the common form of type 2 diabetes (i.e., associated with insulin resistance, hyperinsulinemia, and/or the metabolic syndrome) and it has emerged as a critical therapeutic target in the clinical management of diabetes for the secondary prevention of macrovascular diseases [39]. About 25.6 million (11.3%) adults aged 20 years and older were estimated to have diabetes in the United States in 2010, with type 2 diabetes accounting for about 90% to 95% of all diagnosed cases of diabetes [40]. The clinical guidelines recommend aggressive treatment of dyslipidemia to reduce risk of cardiovascular morbidity and mortality in patients with diabetes [1,41]. American Diabetes Association guidelines recommend lowering LDL-C below 100 mg/dL (i.e., 2.6 mmol/L) as the primary goal of therapy for adults [41,42]. In addition, the NCEP ATP III proposes non-HDL-C as a secondary target of therapy among people with high triglycerides concentration (i.e., ≥ 200 mg/dL). The treatment goal for non-HDL-C is set at 30 mg/dL higher than that for LDL cholesterol [1]. Our results that people with a serum non-HDL-C concentration of 190 mg/dL or above had a greater than two-fold increased risk of death from CVD as those with a concentration below 130 mg/dL provide further support on the use of 190 mg/dL as clinically important threshold value of non-HDL-C concentration in association with excess risk of death from CVD and 130 mg/dL as a therapeutic goal.

Our results of subgroup analyses highlight the consistency in the association between serum non-HDL-C concentration and risk of death from CVD across each level of most demographic variables and selected CVD risk factors. It is noteworthy, however, that the strength in the association between non-HDL-C and risk of death from CVD may vary by sex, age, diabetes duration, length of follow-up, and alcohol consumption because our data clearly demonstrated that there was a significant interaction between these variables and non-HDL-C on the risk of death from CVD. Men have a higher serum non-HDL-C concentration than women in the general population [43]. However, among people with diagnosed diabetes, men have a lower serum non-HDL-C concentration than women, perhaps attributable in part to a higher proportion of men using prescribed medicine to lower blood cholesterol as shown in our study. The variations in the mean serum non-HDL-C concentration and its association with risk of death from CVD between men and women suggest that different cutoff values of non-HDL-C may be needed in clinical practice and research.

It is also worth commenting on the variations in the association between non-HDL-C and risk of death from CVD across age, diabetes duration, and follow-up length in the cohort. Our results that the associations appeared to be stronger among people aged 20 to 64 years than those aged 65 years and older, among people with a diabetes diagnosis less than 10 years than those with diabetes 10 years and above, and among those with a follow-up length 10 years and above than those with a follow-up length less than 10 years all indicate that the effect of serum non-HDL-C on the risk of CVD mortality may be related to a prolonged exposure. In addition, these variations may be helpful in explaining the differences in the findings of previous studies that differ in demographic characteristics and follow-up length [13,14].

Our results are subject to several limitations. First, because only a single measurement of serum non-HDL-C concentration at baseline was available, bias from regression toward the mean may have led us to underestimate the strength for the associations. Second, the diagnosis of diabetes was ascertained by participants' self-report and there could be some misclassification biases. According to a previous study, the agreement between self-report and medical record was substantial (kappa = 0.76) and the sensitivity was high (75%) for diabetes diagnosis [44]. Thus, the impact of the misclassification bias in diabetes status on our results could be minimal. Third, because serum LDL-C concentration was estimated using a subsample of adults who had fasted at least eight hours, the sample size for comparing the relative risk of death from CVD between non-HDL-C and LDL-C was relatively small. Future research using a larger sample may be needed to confirm our results that non-HDL-C performed similarly to LDL-C in relation to CVD mortality.

In conclusion, our findings, based on data from a large representative sample of U.S. adults with diabetes, showed that higher serum non-HDL-C concentrations were significantly associated with the increased risk of death from CVD, particularly from IHD and stroke. They also showed that the positive association was independent of traditional and emerging CVD risk factors. Non-HDL-C may be a more useful target in this population than LDL-C as it reflects all apolipoprotein B100-containing atherogenic lipid particles, it can be assessed at all levels of triglyceride, and it can be measured without fasting. Clinical trials in those with diabetes should consider non-HDL-C as a primary or secondary endpoint.

## List of abbreviations

BMI: Body mass index; CHD: Coronary heart disease; CI: Confidence interval; CKD-EPI: Chronic Kidney Disease Epidemiology Collaboration; CRP: C-reactive protein; CVD: Cardiovascular disease; GFR: Glomerular filtration rate; HDL-C: High-density lipoprotein cholesterol; IDL: Intermediate-density lipoprotein; IHD: Ischemic heart disease; LDL: Low-density lipoprotein; NCEP ATP III: Third Report of the National Cholesterol Education Program Expert Panel on Detection, Evaluation, and Treatment of High Blood Cholesterol in Adults; NCHS: National Center for Health Statistics; NDI: National Death Index; NHANES: National Health and Nutrition Examination Survey; Non-HDL-C: Non-high-density lipoprotein cholesterol; VLDL: Very-low-density lipoprotein.

## Competing interests

The authors declare that they have no competing interests.

## Authors' contributions

All authors fulfill the criteria for authorship. CL and ESF had full access to all the data in the study and take responsibility for the integrity of the data and the accuracy of the data analysis. CL conducted statistical analyses and drafted manuscript. CL, ESF, JT, GZ, LSB, and SSG contributed to study conception and design, interpretation of data, and critical revision of manuscript for important intellectual content. All authors have read and approved submission of the final draft.

### Disclaimer

*The findings and conclusions in this report are those of the authors and do not necessarily represent the official position of the Centers for Disease Control and Prevention*.
